# Supplementary material for: Cl-out is a novel cooperative optogenetic tool for extruding chloride from neurons
Source: Nat Commun. 2016 Nov 17;7:13495. doi: 10.1038/ncomms13495 (PMC5118542; doi:10.1038/ncomms13495)
Supplement: Supplementary Information — Supplementary Figures 1-5 [file ncomms13495-s1.pdf]

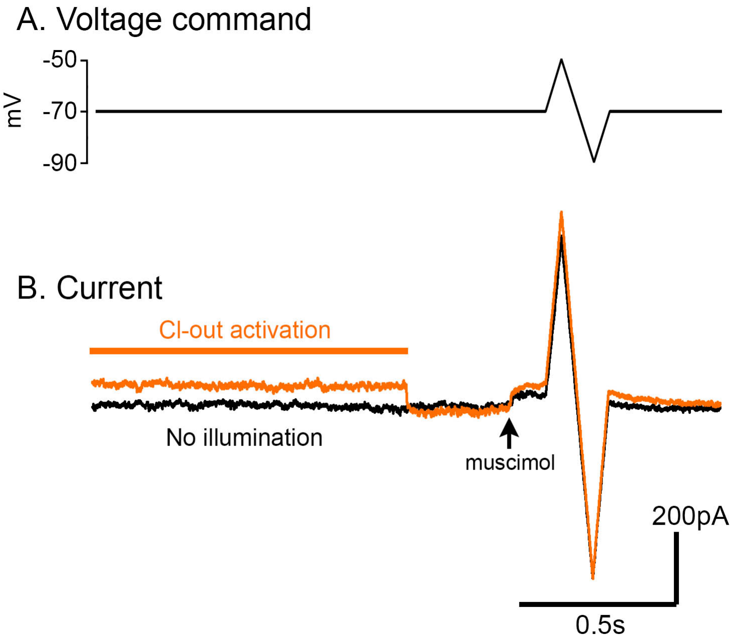

Supplementary figure 1. Voltage ramp response. Example traces showing the full current deflections during the voltage ramps (upper trace, range -50 to -90mV) for two trials, one with Cl-out activation (orange) and one without (black), range -50 to -90mV) for two trials, one with Cl-out activation (orange) and one without (black).

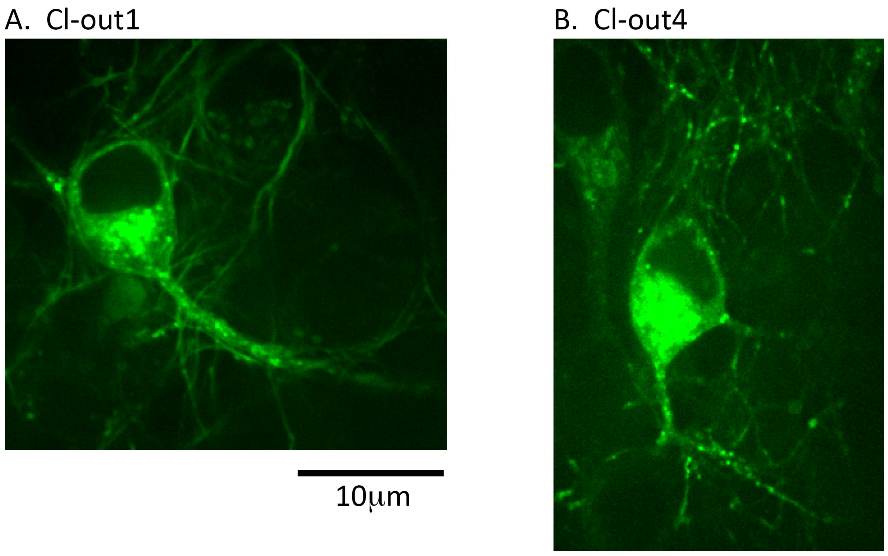

Supplementary figure 2. Membrane localization of Cl-out constructs.
(A) Confocal micrograph of a cultured neuron transfected with the Cl-out1 construct, showing intense EYPF signal throughout the cell, into all dendrites. (B) Similar view of a Cl-out4 transfected cultured neuron.

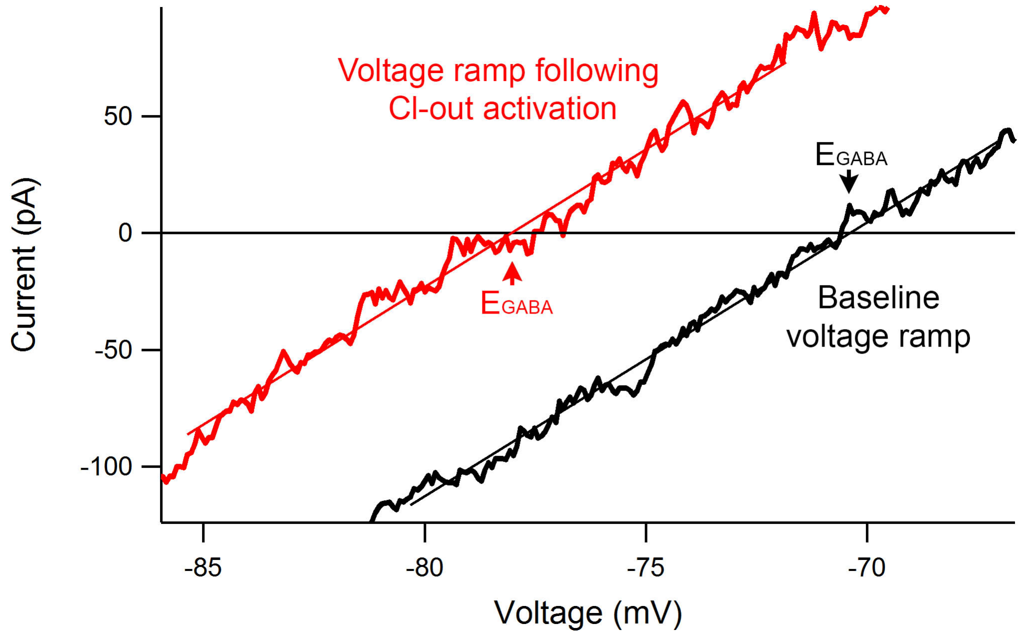

Supplementary figure 3. Cl-out induces changes in E_{GABA}, but not the GABAergic conductance. Plots showing the current during voltage ramps, applied during a response to muscimol during a baseline recording (black) or immediately following a 2s period of Cl-out1 activation. There is a marked shift towards a more negative value of E_{GABA} following Cl-out1 activation, but note that there is no change in the slope of the I-V plot, indicating that there is no change in GABAergic conductance associated with Cl-out1 activation.

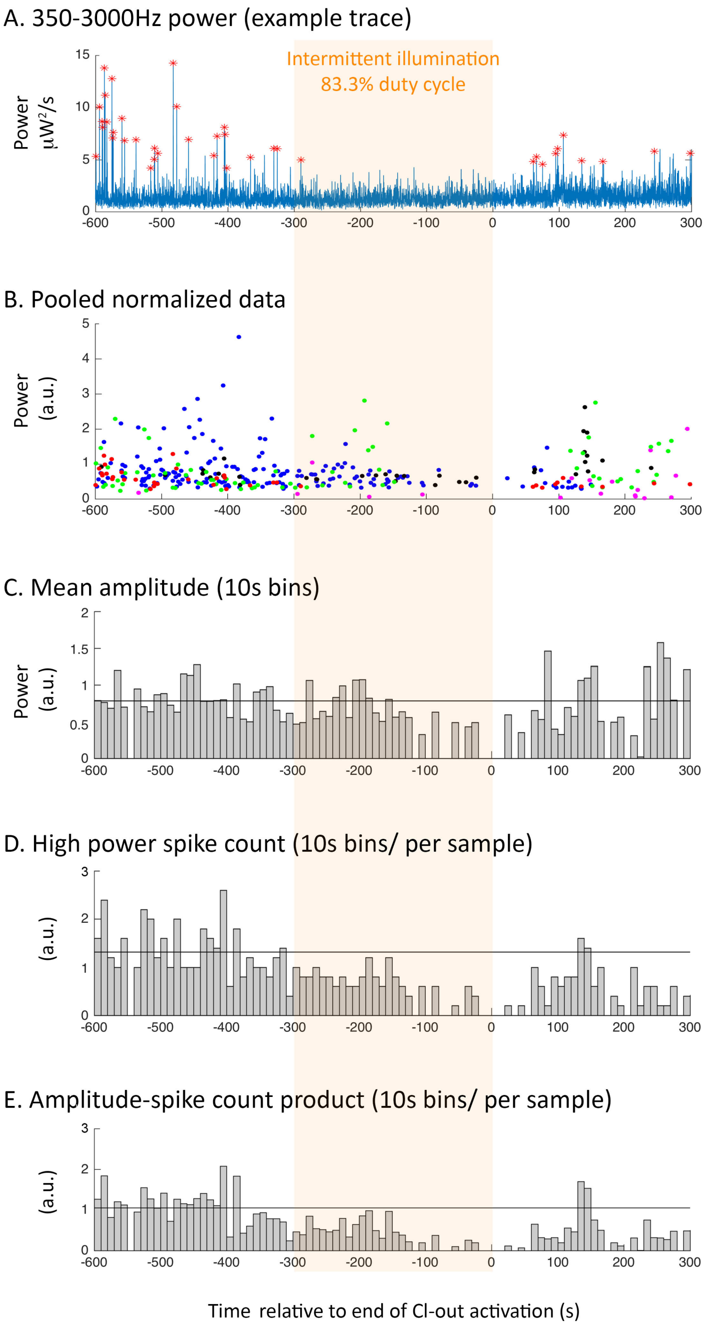

Supplementary figure 4. Protracted, post-illumination effects of Cl-out on spontaneous epileptiform discharges. (A) Example trace of fluctuations in the high frequency component (350-3000Hz) of the extracellular field recording, taken from the pyramidal cell layer of CA3 in a brain slice bathed in VU0473271 (see examples in figures 5 and 6). The high frequency power is sampled over 0.25s bins, and time shifted at 0.05s, which captures the intermittent bursts of epileptiform discharges as sharp peaks in the power trace. The intermittent electrical stimuli also gave sharp peaks, so we removed 500ms epochs at the time of each stimulus for the analysis of spontaneous events, shown here. The lowest activity in all traces came immediately after the end of the period of illumination, so we used this as the baseline measure: baseline epoch, 1-10s postillumination (excluding the response to electrical stimulation that occurred

100ms after the final illumination). Threshold for detection of peaks was set at 4x the standard deviation of the power trace in the baseline epoch. (B) Pooled data from 5 recordings (colour-coded by sample, prepared from 4 animals), normalized to the mean amplitudes of the peaks over the pre-illumimation period. (C) Mean amplitudes in 10s bins. (D) Spike count in 10s bins. (E) Spike x amplitude product for the same 10s bins. Note that all three indices (C-E) decrease during the period of illumination, and then, importantly, remain depressed for tens of seconds after the final illumination cycle.

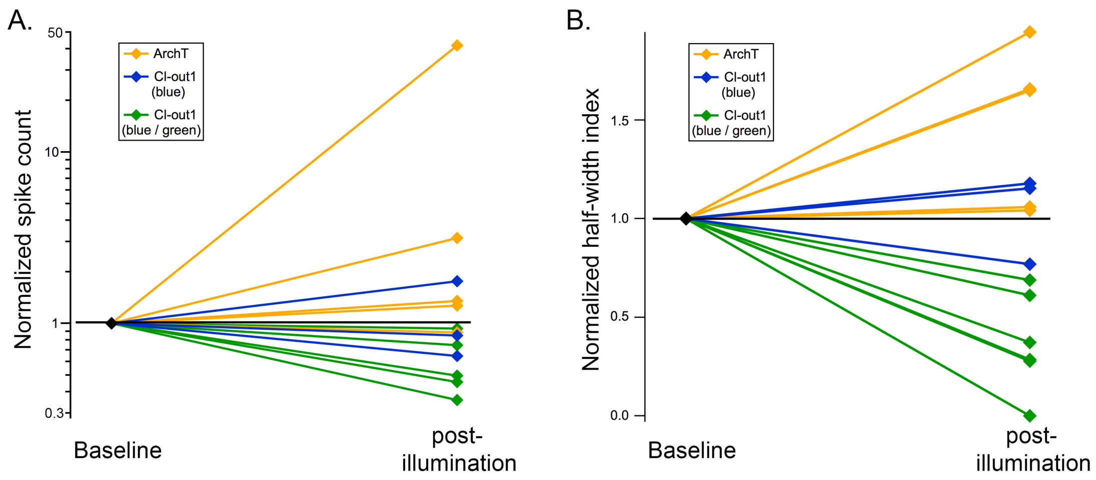

Supplementary figure 5. Comparison of effects on spike count (A) and spike time ("half-width index") for Cl-out1 (green, 6 slices from 4 animals), ChloC (blue, 3 slices from 2 animals) and ArchT (orange, 5 slices from 2 animals). The protocol follows that described in Alfonsa *et al.* (2015). Only activation of both opsins together in the Cl-out construct reduced the spike count and the half-width index.
